# CXCR6 and its correlated G protein-coupled receptors and PI3K-AKT pathways were associated with the progression of thyroid cancer

**DOI:** 10.1186/s12885-026-16513-1

**Published:** 2026-07-10

**Authors:** Yiting Yao, Linlang Huang, Rui Xia, Yang Yuan, Yu Shen, Xingyu Zou, Chenxi Zhao, Chen Chen, Dexuan Chen

**Affiliations:** Affiliated Hospital of Nanjing University of Chinese Medicine, Nanjing, 210029 China

**Keywords:** CXCR6, Thyroid cancer, Prognostic gene, PI3K-AKT, Src, G protein-coupled receptors

## Abstract

**Background:**

Immunogenic cell death (ICD) is closely linked to thyroid cancer (THCA). We mined ICD-linked prognostic genes for survival predictors and unpacked their potential biology.

**Materials:**

Single-cell analysis was conducted to identify distinct cell types, and the scores of ICD-related genes (ICDRGs) were computed to identify key cell types.

Furthermore, correlation, differential expression, and Kaplan-Meier survival studies were conducted to discover prognostic genes. By employing these genes alongside 101 combinations of 10 algorithms, we constructed a prognostic model and computed risk scores for THCA samples, analyzing their survival disparities. Control and THCA tissue samples were taken to assess the expression of prognostic genes via real-time reverse transcriptase-polymerase chain reaction (RT-qPCR), resulting in the identification of a key gene. Subsequently, gene set enrichment analysis was executed on this key gene, and Western blot analysis was undertaken to evaluate the expression of relevant pathways.

**Results:**

Single-cell and transcriptome analysis found CXCR6, ICOS, SLAMF7, CD80, and TNF as prognostic genes associated with ICD in THCA. Of the 101 combinations evaluated, the StepCox[both] + Random Survival Forest (RSF) model was selected as the prognostic model owing to its superior C-index. This model accurately forecasted survival outcomes for THCA samples. Expression study demonstrated a notable decrease of CXCR6 in THCA samples, consistent with bioinformatics analysis and it was designated as the key gene. Functional analysis revealed an enrichment of the inflammatory response, accompanied by reduced levels of its principal targets (Gαi) and downstream pathways (Src and PI3K-AKT) in THCA samples.

**Conclusion:**

This study found five prognostic genes, with CXCR6 associated with Gαi, Src, and PI3K-AKT pathways in the modulation of THCA, indicating the essential function of CXCR6 in THCA.

**Supplementary Information:**

The online version contains supplementary material available at https://doi.org/10.1186/s12885-026-16513-1.

## Introduction

Thyroid cancer (THCA) is a malignant tumour originating from the epithelial cells of the thyroid follicles and represents the most common endocrine malignancy [1].

THCA encompasses various pathological subtypes, including various pathological subtypes including papillary carcinoma, follicular carcinoma, medullary carcinoma, and anaplastic carcinoma [2]. Among these, thyroid papillary carcinoma (PTC) is the predominant histological subtype, accounting for approximately 85% of all THCA cases [2]. In recent decades, the global incidence of THCA has shown a steady upward trend, but the mortality rate remains relatively low due to the indolent nature of most subtypes [3]. Currently, the main treatment strategies for THCA include surgical resection, radioactive iodine therapy, and targeted therapy [4]. However, for advanced or metastatic THCA, especially anaplastic THCA, the therapeutic effect is often unsatisfactory, with high recurrence rate and poor prognosis [5, 6]. The lack of reliable prognostic biomarkers and effective therapeutic targets remains a major challenge in the clinical management of THCA. Therefore, identifying novel genes associated with the progression and prognosis of THCA is crucial for improving therapeutic outcomes.

Immunogenic cell death (ICD) is a special form of programmed cell death that can trigger adaptive immune responses by releasing immunogenic signals such as damage-associated molecular patterns (DAMPs) [7]. ICD plays a dual role in tumor progression: it can not only directly eliminate tumor cells but also regulate the tumor immune microenvironment to enhance anti-tumor immunity [8]. As a critical immune regulatory process, ICD recognizes DAMPs released by dying cells within the tumor microenvironment, thereby activating innate immune responses and initiating tumor-specific adaptive immunity [9]. Substantial preclinical and clinical evidence indicates that the therapeutic efficacy of various effective anticancer treatments is closely linked to their ability to induce ICD in tumor cells [10]. Recent studies have further revealed that ICD-related genes hold significant prognostic value in cancer, with prognostic models based on ICDGs successfully constructed for multiple malignancies including head and neck squamous cell carcinoma, low-grade glioma, and ovarian cancer [11]. Collectively, these findings suggest that the capacity to induce immunogenic cell death in tumor cells is a key determinant of treatment outcomes and patient prognosis. Although some studies have explored the association between ICD and THCA [12], the specific mechanism by which ICD regulates THCA progression and the identification of related prognostic genes remain unclear, urgently requiring further investigation.

In this study, we integrated single-cell and bulk RNA sequencing data to identify key cell types and ICD-related genes associated with THCA prognosis. Through comprehensive analyses including pseudotime trajectory, cell communication, differential expression, and survival modeling using multiple machine learning algorithms, we developed a robust prognostic model. Experimental validation by RT-qPCR and functional exploration via Gene set enrichment analysis (GSEA) and Western blot further elucidated the molecular mechanisms of key ICD-related genes. Our findings provide novel prognostic biomarkers and potential therapeutic targets for THCA.

## Materials and methods

### Data source

The transcriptomic bulk RNA-seq dataset of THCA (TCGA-THCA) was obtained from the UCSC Xena (https://xenabrowser.net/datapages/). The TCGA-THCA cohort contained 500 thyroid carcinoma tissues and 57 pathologically annotated normal thyroid control tissues, among which 499 tumor samples possessed complete survival clinical data. The single-cell RNA sequencing (scRNA-seq) dataset GSE191288 was obtained from the Gene Expression Omnibus (GEO) databases (https://www.ncbi.nlm.nih.gov/gds/), consisting of 6 THCA specimens and 1 normal thyroid control sample. A total of 32 immunogenic cell death-related genes (ICDRGs) were extracted from the previously published study by Dai et al. [13]. We adopted the complete curated ICD gene set from this literature without additional screening, and the full list of all 32 ICDRGs was summarized in Table S1.

### Single-cell analysis

In GSE191288 dataset, quality control (QC)was conducted on data using “Seurat” package (version 5.1.0) [14] with following eliminated thresholds: (1) genes detected in less than 200 cells; (2) cells with nFeature ≥ 9,000, nCount ≥ 65,000, and mitochondrial genes ≥ 20%. After QC filtering, global log-normalization was implemented using the NormalizeData function with a scale factor of 10,000. Next, the FindVariableFeatures function with the “vst” algorithm was applied to identify the top 2,000 highly variable genes for downstream dimensionality reduction. The Harmony algorithm was utilized to eliminate cross-sample batch effects among specimens. Principal component analysis was carried out, and the first 30 principal components were retained for subsequent clustering. The FindNeighbors function (dims = 1:30) and FindClusters function with a resolution of 0.3 were applied for unsupervised cell clustering. Nonlinear dimensional reduction and visualization were performed via UMAP with customized parameters: n.neighbors = 50 and min.dist = 0.5. Cell type annotation was achieved using the FindAllMarkers function [15], and all canonical marker genes corresponding to each annotated cell subtype have been summarized in Table S2. To reduce the influence of doublets, “DoubletFinder” package (version 2.0.4) [16] was used to identify and remove them. The proportion of cell types was further evaluated in control and THCA samples. Furthermore, ICDRGs-related scores of each cell type were calculated by 5 algorithms-AUCell, UCell, singscore, ssGSEA, and AddModuleScore. To avoid bias introduced by a single algorithm, we normalized the scores generated by each of the five tools separately and calculated their average value as the comprehensive ICDRG score of each individual cell. Cell types with the highest average comprehensive ICDRG scores were defined as key ICD-related cell types. This analytical step was designed to identify cell populations with prominent ICD pathway activation rather than statistically evaluating the association between ICD scores and tumor progression, hence relevant statistical tests were not performed here. Key cell types were determined with the highest average scores. Pseudotime analysis was then conducted on key cell types to explore their differentiation states and the expression of ICDRGs throughout differentiation through “Monocle” package (version 2.26.0) [17]. The interactions among cell types were explored by cell communication analysis through “CellChat” package (version 1.6.1) [18]. The correlation between average ICDRG scores of key cell types and ICDRGs was analyzed, and genes with cor > 0.1 and *P* < 0.05 were retained as signature genes for further analysis.

Additionally, “Scissor” package (version 2.0.0) [19] was used to identify THCA survival-related cells (Scissor+: positive correlation, Scissor-: negative correlation) based on the integration of bulk RNA and scRNA-seq datasets. Differential expression analysis was then performed between Scissor + and Scissor- cell types to identify differentially expressed genes 1 (DEGs1) with log_2_FC ≥ 0.5, min.pct ≥ 0.25, and *P* < 0.05.

### Identification of differentially expressed-ICDRGs (DE-ICDRGs)

In TCGA-THCA dataset, ICDRG scores of THCA samples were calculated by ssGSEA algorithm in “GSVA” package (version 1.46.0) [20]. Samples were stratified into high- and low-score subsets, followed by survival comparison using Kaplan-Meier (KM) estimation via “survival” (version 3.4-0) [21] and “survminer” packages (version 0.4.9) [22]. Differential expression analysis was then conducted between score groups to identify DEGs2 using “DESeq2” package (version 1.38.3) [23] with |log_2_FC| > 0.5 and *P* < 0.05. The volcano plot of DEGs2 and heat map of top 10 up- and down-regulated DEGs2 were respectively drafted by “ggplot2” (version 3.5.1) [24] and “pheatmap” packages (version 1.0.12) [25]. DEGs1, DEGs2, and signature genes were intersected to determine DE-ICDRGs, which were visualized by “ggvenn” package (version 0.1.10) [26]. GO and KEGG enrichment analyses were carried out by “clusterProfiler” package (version 4.6.2) [27] with adj. *P* < 0.05.

### Identification of prognostic genes in THCA

In TCGA-THCA dataset, the expression of DE-ICDRGs was explored between control and THCA samples. Genes with significant difference were identified as candidate genes. Using the optimal cut-off generated by surv_cutpoint (“survminer” package), THCA cases were split into high- and low-expression cohorts. Survival curves were plotted with KM estimates and evaluated by the log-rank test; candidates reaching *P* < 0.05 were retained as prognostic genes.

### Development of prognostic model in THCA

In TCGA-THCA dataset, based on stratified sampling implemented via the createDataPartition function in the R “caret” package, all THCA patients were randomly split into training and internal validation subsets at a 6:4 ratio to balance survival status and clinical features between two cohorts. Using the screened ICD-related prognostic genes, we exhaustively combined 10 independent survival algorithms (Least Absolute Shrinkage and Selection Operator (LASSO), Random Survival Forest (RSF), Elastic Net (Enet), Stepwise Cox Proportional Hazards Regression (Stepwise Cox), Ridge Regression (Ridge), Cox Model with Boosting (CoxBoost), Partial Least Squares Regression for Cox Models (plsRcox), Supervised Principal Components (SuperPC), Gradient Boosting Machine (GBM) and Support Vector Machine – Recursive Feature Elimination (SVM-RFE)) to generate 101 distinct model combinations, and C-index was calculated to quantify the predictive performance of each model. The best-performing ensemble, identified by the highest C-index, was adopted as the final prognostic model and used to compute per-sample risk values. ROC analysis gauged its discriminative capacity, and an optimal threshold on these scores split the cohort into high- and low-risk strata. Their survival condition was visualized by KM survival curve, while differences were compared by log-rank test. The overall survival (OS) was used as the sole clinical survival endpoint for machine learning model construction and all KM survival analyses. The capability of prognostic model was verified in validation set. In addition, univariate and multivariate Cox regression analyses were conducted to evaluate the independent prognostic capacity of the risk score. Clinical covariates including age, tumor stage, T stage, and risk score were incorporated into regression models. Forest plots of univariate and multivariate Cox results were generated and presented.

THCA datasets in the GEO database lack complete OS and vital status data and are only suitable for differential expression analysis between tumour and normal tissues, so external cohort validation of the prognostic signature cannot be performed in the present study. We randomly split the TCGA-THCA cohort at a 6:4 ratio to generate an independent internal validation set, whose sample size is comparable to or larger than external validation cohorts used in most analogous THCA prognostic studies. All survival-based model evaluations, including time-dependent ROC curves, C-index and KM OS stratification, were implemented on the validation set without any data leakage, which reliably assessed the predictive robustness of our machine learning model.

### Gene set enrichment analysis (GSEA)

Differential expression analysis was conducted between high and low risk groups, and log_2_FC values were the ranking thresholds. KEGG-oriented (“c2.cp.kegg_legacy.v2025.1.Hs.symbols.gmt”) GSEA was then conducted using “clusterProfiler” package, pathways with |NES| > 1 and *P* < 0.05 were deemed enriched.

### Immune microenvironment and mutation analyses

The proportion of 22 immune cells was computed using “CIBERSORT” package (version 0.1.0) [28], and the disparity across two risk groups was assessed by Wilcoxon test. Correlation between prognostic genes and differential immune cells was analyzed by Spearman analysis. Mutation conditions between two risk groups were evaluated by “maftools” package (version 2.14.0) [29].

### Clinical correlation and drug sensitivity

The correlation between risk scores and clinical factors such as age, stage, gender, and T/N/M stages was explored by Wilcoxon and Chi-square tests. Subsequently, IC_50_ value for different immune checkpoint inhibitors was computed by “oncoPredict” package (version 1.2) [30], and compared between risk groups.

### The expression and prognostic analyses of prognostic genes in pan-cancer

Based on TIMER and GEPIA databases, the expression of prognostic genes between control and cancer groups was analyzed in pan-cancer. According to the optimal threshold of prognostic gene expression, cancer samples were stratified into high and low-expression groups and their survival prognosis (including OS, DSS, PFI, and DFI) was visualized by KM survival curve.

### Tissue sample collection

Six pairs of THCA tissues and corresponding adjacent normal thyroid tissues (≥ 2 cm from the tumor edge, pathologically confirmed free of tumor infiltration) were surgically resected and collected from the Department of General Surgery, Jiangsu Provincial Hospital of Traditional Chinese Medicine, during March 2025 to September 2025. None of the patients had received radiotherapy, chemotherapy, targeted therapy or immunotherapy prior to surgery, and all were diagnosed as THCA by postoperative pathology. Their clinical baseline data were collected simultaneously for subsequent analysis. This study obtained written informed consent from all participants and was approved by the Ethics Review Committee of Jiangsu Provincial Hospital of Traditional Chinese Medicine (Approval No.: 2023NL-287-02). All experimental procedures were performed in compliance with the Declaration of Helsinki and relevant ethical guidelines. Following sterile processing, the resected tissue samples were snap-frozen in liquid nitrogen and stored at -80℃ for subsequent RNA extraction and Western blot analysis. All tissue snap-freezing, RNA and protein extraction operations followed standardized tissue processing protocols reported in comparable translational tissue research systems [31, 32].

### Determination and analysis of key genes

RNA from tissue samples was isolated with FastPure Complex Tissue/Cell Total RNA Isolation Kit (Vazyme, RC113-01), quantified on a Nanodrop-500 (Allsheng, Nano500), and reverse transcribed using ABScript III RT Master Mix for RT-qPCR with gDNA Remover (Abclonal, RK20429). RT-qPCR was run with Genious 2X SYBR Green Fast RT-qPCR Mix (Abclonal, RK21205). The sequences for the primers were displayed in Table 1. The mRNA expression was normalized to GAPDH, and calculated by 2^−ΔΔCt^ approach. Genes whose trends matched the bioinformatics data were retained as key genes. Then, correlation coefficient of key genes and all genes were ranking criteria. Through “clusterProfiler” package, GSEA was performed with |NES| > 1 and adj.*P* < 0.05 based on background gene set “h.all.v2025.1.Hs.symbols.gmt”, downloaded from GSEA website.

**Table 1:** The sequences for primers used in Real-time reverse transcriptase-polymerase chain reaction (RT-qPCR)

Primers	Sequences 5’-3’
GAPDH	F: TGGTGAAGGTCGGTGTGAAC
	R: TTCTCAGCCTTGACTGTGCC
CXCR6	F: TCAATGACAGCAGCCAGGAG
	R: GTGTCATCTGGGTGGCAAGA
ICOS	F: CTTTCTCTTCTGCTTGCGCA
	R: TTGGCTGTGTTCACTGCTCT
SLAMF7	F: CCAACATGCCTCACCCTCAT
	R: ATAGCCTTGGTGTGTCTGGC
CD80	F: CCGAGTACAAGAACCGGACC
	R: CTCTCTGCATCTTGGGGCAA
TNF	F: CCTCTCTCTAATCAGCCCTCTG
	R: GAGGACCTGGGAGTAGATGAG

### Western blot

Total proteins were extracted from tissue samples using RIPA lysis buffer (Beyotime, P0013B), containing 1% PMSF (Servicebio, CR2404006), and their concentration was determined by a BCA kit (Beyotime, P0010). Proteins were separated by SDS-PAGE, followed by the transfer to PVDF membranes (Milipore, 0000279048).

These membranes were blocked in 5% non-fat milk (Servicebio, GC310001-100 g) for 30 min, which were treated with primary antibodies overnight at 4℃. Subsequently, membranes were cultured using corresponding secondary antibodies for 30 min.

Protein bands were detected using ECL (Milipore, WBSKLS), and evaluated by Image J software. The whole protein extraction, electrophoresis, membrane transfer and immunoblotting workflow adopted standard tissue protein quantitative procedures consistent with previously reported tissue detection methods [31, 32]. The antibodies used in Western blot were all provided in Table S3.

### Statistical analysis

All bioinformatics analyses were done in R software (version 4.2.2), pairwise contrasts used Wilcoxon test. Experimental data were analyzed in GraphPad Prism software (version 8.0), represented as mean ± standard deviation (SD) and compared by one-way ANOVA. A *P* value of less than 0.05 was considered statistically significant.

## Results

### T cells were identified as key cell types

**Fig. 1:**
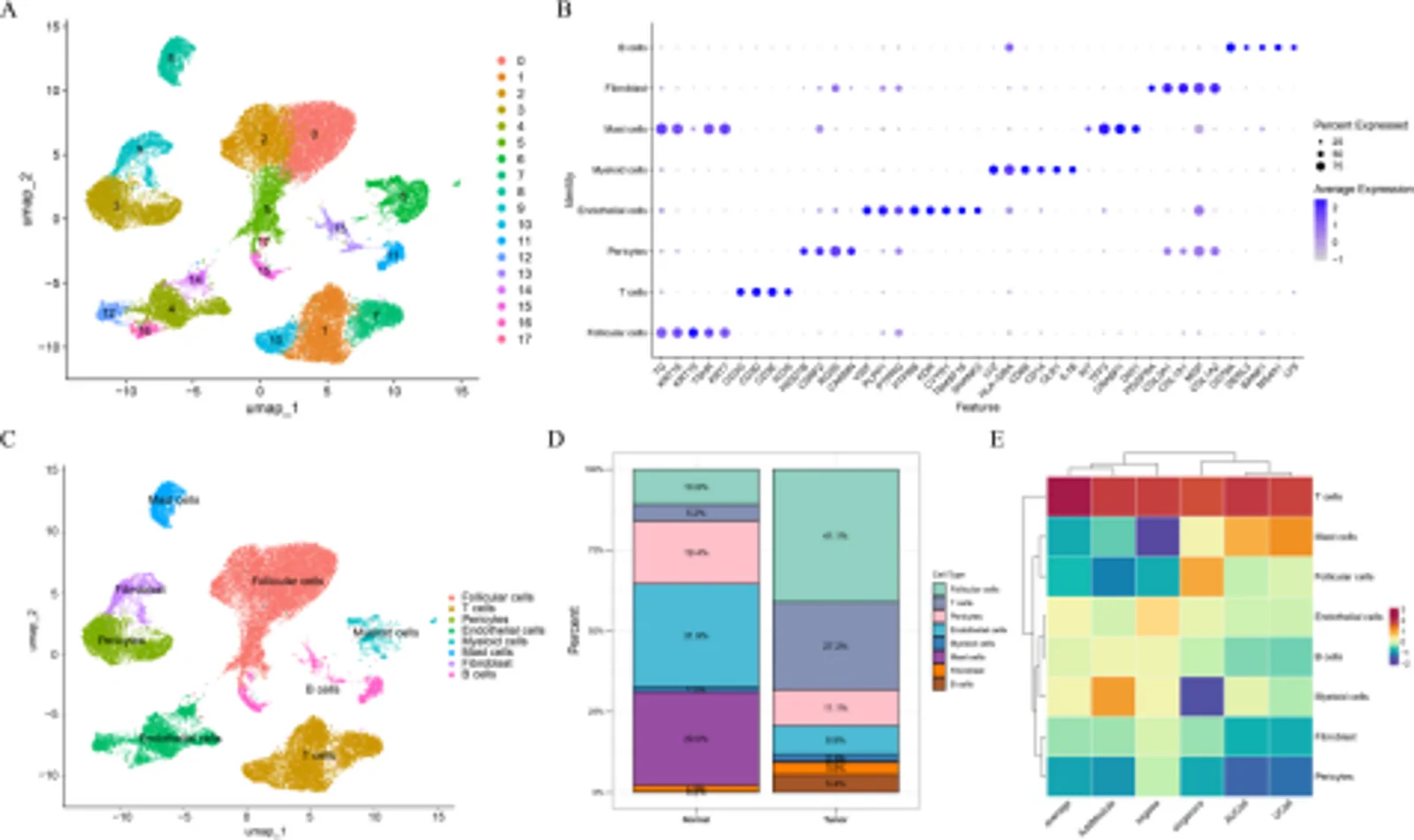
T cells were identified as key cell types. **A **The identification of cell clusters. **B** Marker genes for cell types. **C** The annotation of cell types. **D** The proportions of cell types in control and thyroid cancer (THCA) samples. **E** Immunogenic cell death-related gene (ICDRG) scores for each cell type

To assess the signature in the THCA scRNA-seq dataset, we conducted a comprehensive single-cell analysis. After quality control, 30,254 cells and 30,502 genes were retained (Fig. S1A). Next, 18 cell clusters were identified (Fig. 1A), which were then annotated as 8 cell types, such as follicular, T, endothelial, myeloid, mast, fibroblast, B cells, and pericytes (Fig. 1B, C). A total of 2,269 doublets (7.5%) were removed, and 27,985 singlets were remained with clearer boundaries (Fig. S1B).

Further proportion analysis showed that follicular cells were predominant in THCA, followed by T cells (Fig. 1D). ICDRG scores for each cell type were calculated, and T cells exhibiting the highest scores as key cell types (Fig. 1E).

**Fig. 2:**
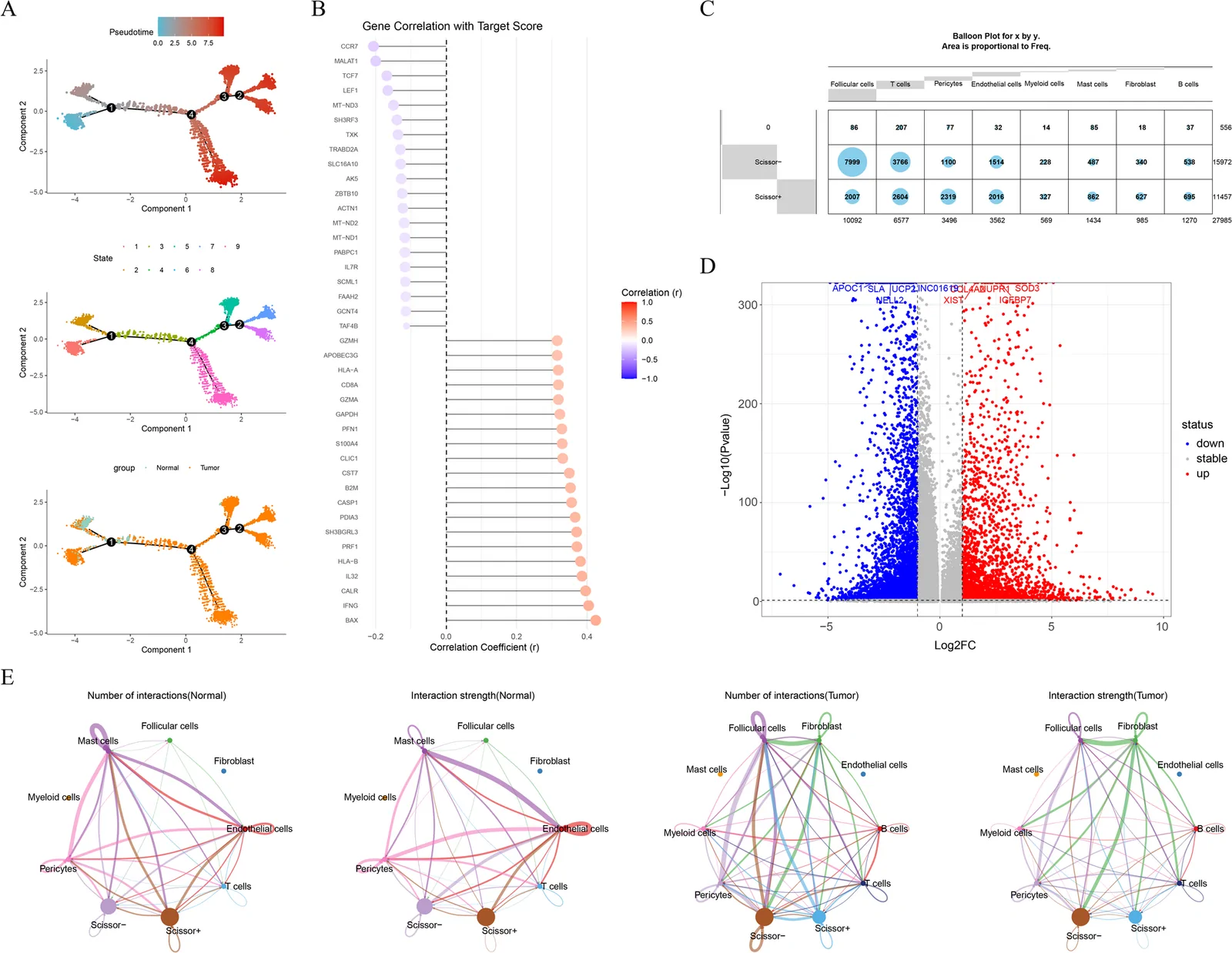
Pseudotime analysis of T cells and differential expression analysis. **A** Pseudotime analysis of T cells. **B** The correlation between ICDRGs and average ICDRG scores of T cells. **C** Cell types positively and negatively correlated with THCA. **D** The volcano plot of differentially expressed genes (DEGs) between positively and negatively correlated cells. **E** Cell communication analysis among cell types

Pseudotime analysis revealed 9 differentiation states of T cells, and all stages were more in THCA samples than control (Fig. 2A). Among 32 ICDRGs, HSP90AA1 had the highest expression during T cell differentiation, followed by the HMGB1 (Fig. S1C). Through correlation analysis between T cell scores and ICDRGs, 2,445 signature genes were screened out with cor > 0.1 and *P* < 0.05 (Fig. 2B). Furthermore, Scissor algorithm showed that all cell types both had positive and negative cells correlated with THCA survival (Fig. 2C). Between Scissor + and Scissor- cells, 14,659 DEGs, including 3,594 up- and 11,065 down-regulated genes, were identified (Fig. 2D). Cell communication analysis indicated that the interactions among some cell types were more and stronger in THCA than control groups, such as follicular cells, fibroblast, and Scissor+ cells (Fig. 2E). This Scissor-based differential gene screening captured transcriptomic disparities between survival-beneficial and harmful cell subpopulations arising from intratumoral heterogeneity, and the obtained DEGs1 were incorporated into cross-dataset intersection analysis to filter reliable ICD-related prognostic genes in the subsequent workflow.

### A total of 29 DE-ICDRGs were identified

**Fig. 3:**
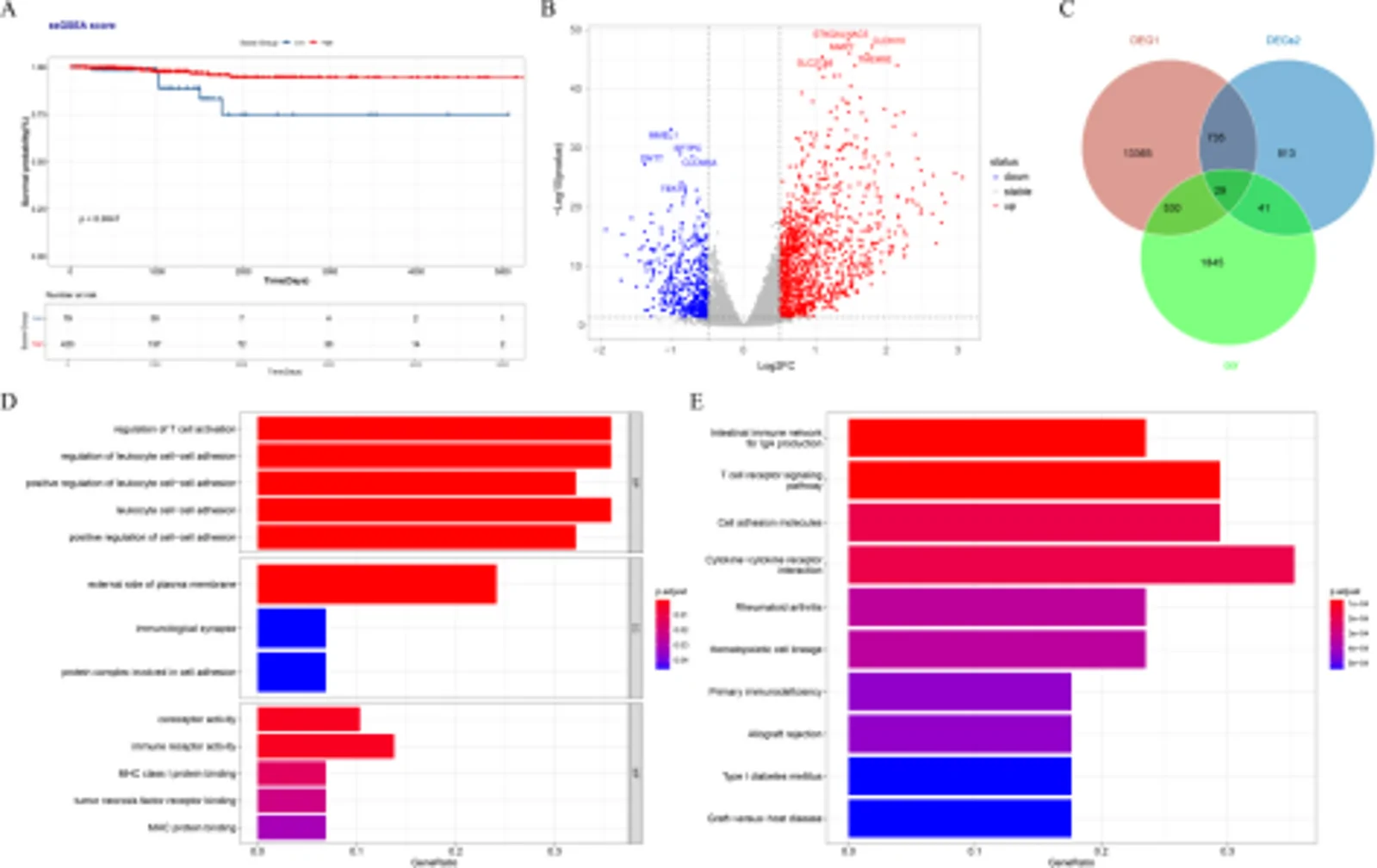
A total of 29 differentially expressed-ICDRGs (DE-ICDRGs) were identified. **A** Kaplan-Meier (KM) overall survival (OS) analysis between ICDRG high and low score groups. **B** The volcano plot of DEGs2 between high and low score groups. **C** The venn diagram of intersection for positively correlated genes, DEGs, and DEGs2. **D-E** Gene ontology (GO) (**D**) and Kyoto Encyclopedia of Genes and Genomes (KEGG) (**E**) enrichment analyses of DE-ICDRGs

TCGA-THCA tumors were split by ICDRG ssGSEA scores, higher scores tracked longer survival on KM plot (Fig. 3A). Differential expression analysis between these score groups revealed 1,618 DEGs2 (1,154 up-regulated and 464 down-regulated) (Fig. 3B). The intersection of 2,445 signature genes, 14,659 DEGs1, and 1,618 DEGs2 yielded 29 intersection genes, namely DE-ICDRGs (Fig. 3C). Enrichment analyses of these DE-ICDRGs revealed the enrichment of 304 GO terms (regulation of T cell activation and immune receptor activity) and 22 KEGG pathways (cytokine-cytokine receptor interaction and T cell receptor signaling pathway) (Fig. 3D, E).

### CXCR6, ICOS, SLAMF7, CD80, and TNF were identified as prognostic genes

**Fig. 4:**
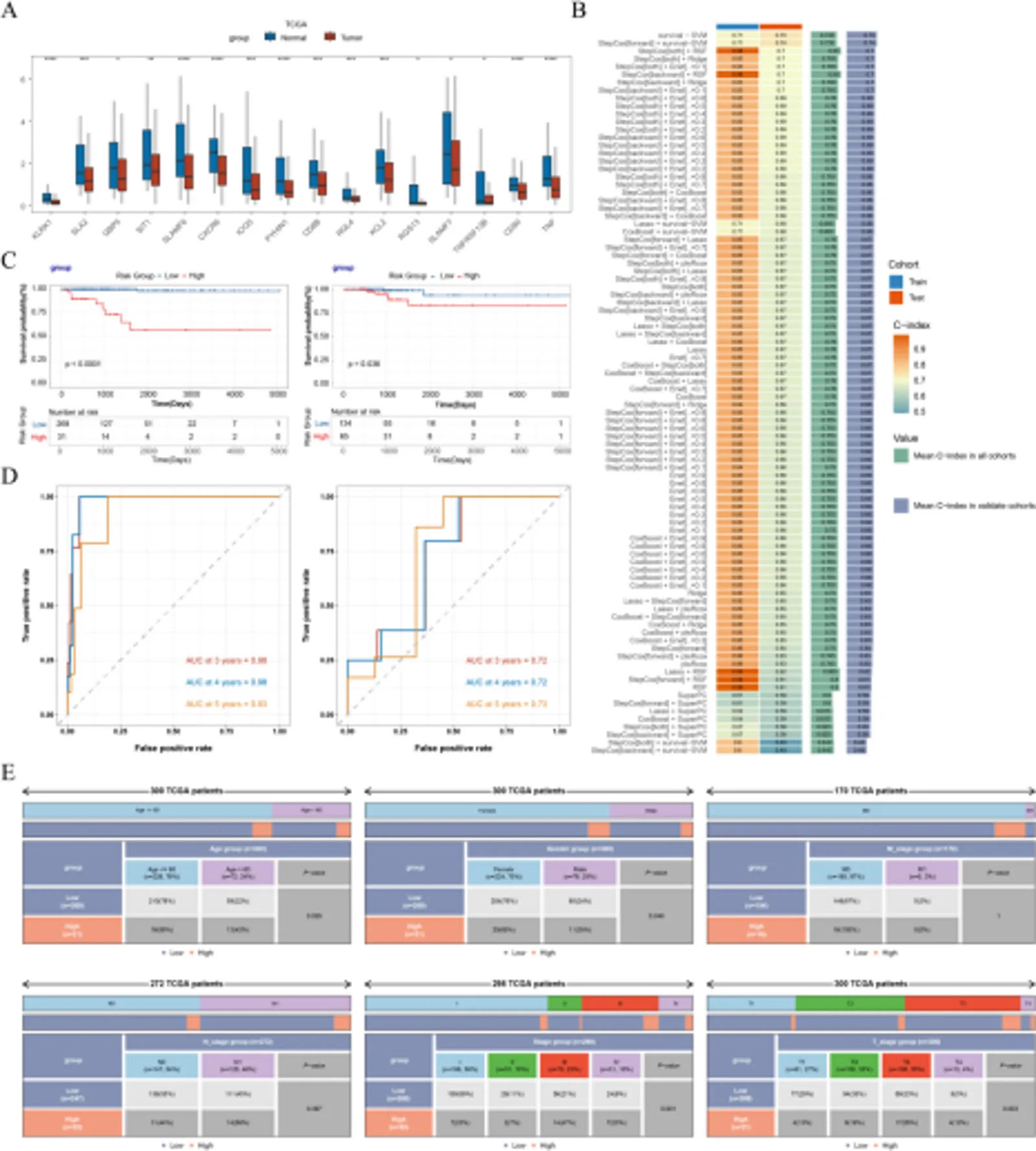
CXCR6, ICOS, SLAMF7, CD80, and TNF were identified as prognostic genes. **A** The differential expression of DE-ICDRGs between THCA and control samples. **B** Selection of optimal model as prognostic model with C-index value. **C** KM survival analysis between high and low risk groups in both training and validation sets of THCA transcriptome dataset. **D** Receiver operating characteristic (ROC) curve for prognostic model performance evaluation. **E** The correlation between risk scores and clinical factors. *P < 0.05, **P < 0.01, ***P < 0.001, ****P < 0.0001. AUC: area under the curve

Of these 29 DE-ICDRGs, 16 exhibited significantly differential expression between THCA and control samples (Fig. 4A). We further stratified all TCGA-THCA patients into high- and low-expression subgroups according to the optimal cutoff value of each gene, and Kaplan–Meier OS analysis revealed markedly distinct survival outcomes between subgroups for CD80, CXCR6, ICOS, SLAMF7, and TNF (Fig. S2A). These five genes were therefore identified as ICD-related prognostic genes for THCA. We adopted a stratified sampling strategy to randomly divide the entire TCGA-THCA cohort into training and internal validation sets at a 6:4 ratio to maintain balanced clinical and survival characteristics across two subgroups. Using the five screened prognostic genes, we exhaustively combined 10 machine learning algorithms to generate a total of 101 distinct prognostic model combinations, and the C-index metric was used to quantify predictive performance in both cohorts. The StepCox[both] + RSF ensemble model was selected as the final optimal prognostic signature, as it achieved the maximum C-index in the training cohort while retaining robust predictive performance in the independent validation set and outperformed the remaining 100 algorithm combinations (Fig. 4B). Specifically, the C-index reached 0.98 (95% CI: 0.95–0.99) in the training cohort and 0.70 (95% CI: 0.55–0.86) in the validation cohort. The optimal risk score cutoff value of 0.71 was determined and uniformly applied to both training and validation cohorts to dichotomize all THCA samples into high-risk and low-risk subgroups. Time-dependent ROC curves were applied to evaluate the discriminative ability of this signature, and the AUC values were all greater than 0.7 in both datasets (Fig. 4C). We calculated the risk score for each patient based on the integrated model, and Kaplan–Meier survival curves demonstrated that patients in the low-risk subgroup possessed significantly prolonged overall survival in both training and validation cohorts (Fig. 4D). Subsequent clinical correlation analysis indicated that risk scores were significantly correlated with clinical characteristics including patient age, tumor stage, and T stage (Fig. 4E).

Pan-cancer expression analysis showed that all five prognostic genes exhibited significant differential expression between tumor and normal tissues in ESCA and HNSC (Fig. S2B). Moreover, KM survival curves illustrated significant discrepancies in OS, DSS, PFI, and DFI between high- and low-expression groups of these prognostic genes across multiple malignancies, including OV and UCEC (Fig. S3). To verify whether the risk score derived from the StepCox[both] + RSF model could serve as an independent prognostic biomarker, we performed univariate and multivariate Cox proportional hazards regression analyses incorporating common clinical variables, including age, tumor stage, T stage, and risk score. Univariate Cox regression analysis revealed that the risk score was significantly associated with patient OS (Fig. S4A). After adjusting for all confounding clinical factors in multivariate Cox regression, the risk score retained statistical significance as an independent prognostic indicator for THCA patients (Fig. S4B). These results suggested that our ICD-related prognostic signature possessed independent predictive power for overall survival, regardless of routine clinical pathological characteristics.

### Immune, drug sensitivity, and mutation analyses in THCA samples with diverse risk

**Fig. 5:**
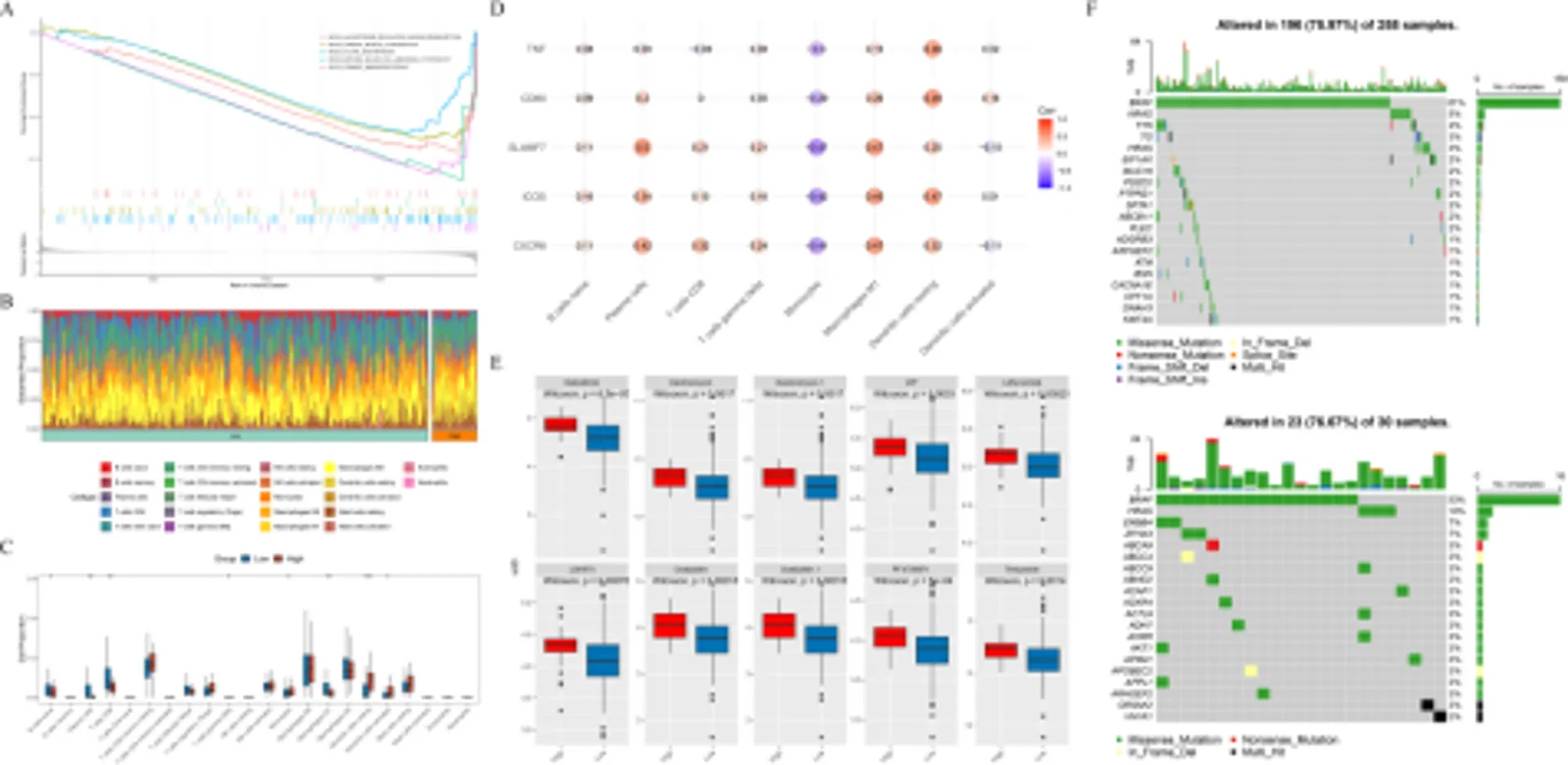
Immune, drug sensitivity, and mutation analyses in THCA samples with diverse risk. **A** The functional enrichment in high and low risk groups. **B** Immune cell infiltration profiles in high and low risk groups. **C** The difference of immune cells between high and low risk groups. **D** Correlation analysis between differential immune cells and prognostic genes. **E** The differential drug sensitivity between high and low risk groups. **F** Mutation conditions in both high and low risk groups. *P < 0.05, **P < 0.01, ***P < 0.001

To explore the functional pathways in THCA development, GSEA pinpointed 15 significantly enriched pathways (Fig. 5A). Top 5 of these pathways were aldosterone regulated sodium reabsorption, cardiac muscle contraction, folate biosynthesis, natural killer cell mediated cytotoxicity, and primary immunodeficiency. Notably, primary immunodeficiency described the genetic mutations causing primary immunodeficiency disorders and their effects on molecular signaling networks. Due to the significant role of immunity in THCA, immune infiltration analysis was conducted and immune cell profiles were displayed in Fig. 5B. Marked differences were existed in 8 types, such as naive B cells, plasma cells, CD8 T cells and monocytes between THCA and control samples (Fig. 5C). Correlation analysis further demonstrated that the highest positive and negative correlations were existed in SLAMF7 with plasma cells (cor = 0.5, *P* < 0.05) and monocytes (cor = -0.47, *P* < 0.05), respectively (Fig. 5D). These results indicated that different immune conditions in THCA samples with various risk. Among 198 common drugs, 83 types exhibited significant difference in IC_50_ value across diverse risk groups. Top 10 drugs ranked by *P* value included dabarfenib (Fig. 5E), which has been used in the treatment of THCA.

This drug had lower IC_50_ value in low risk group, suggesting higher sensitivity for dabarfenib in patients in this group. Further mutation analysis illustrated that BRAF was the gene with the highest mutation rate in both risk groups (high risk: 53%, low risk: 61%) (Fig. 5F). Combined with mutation profiling revealing BRAF as the most prevalent driver mutation across risk groups, the differential dabrafenib sensitivity results provide translational clinical reference: low-risk THCA patients with BRAF mutation may achieve better therapeutic response to dabrafenib plus trametinib combination regimens, supporting individualized clinical medication guided by our ICD-derived risk signature.

### The control of THCA by CXCR6 was associated with the Gαi, Src, and PI3K-AKT pathways

**Fig. 6:**
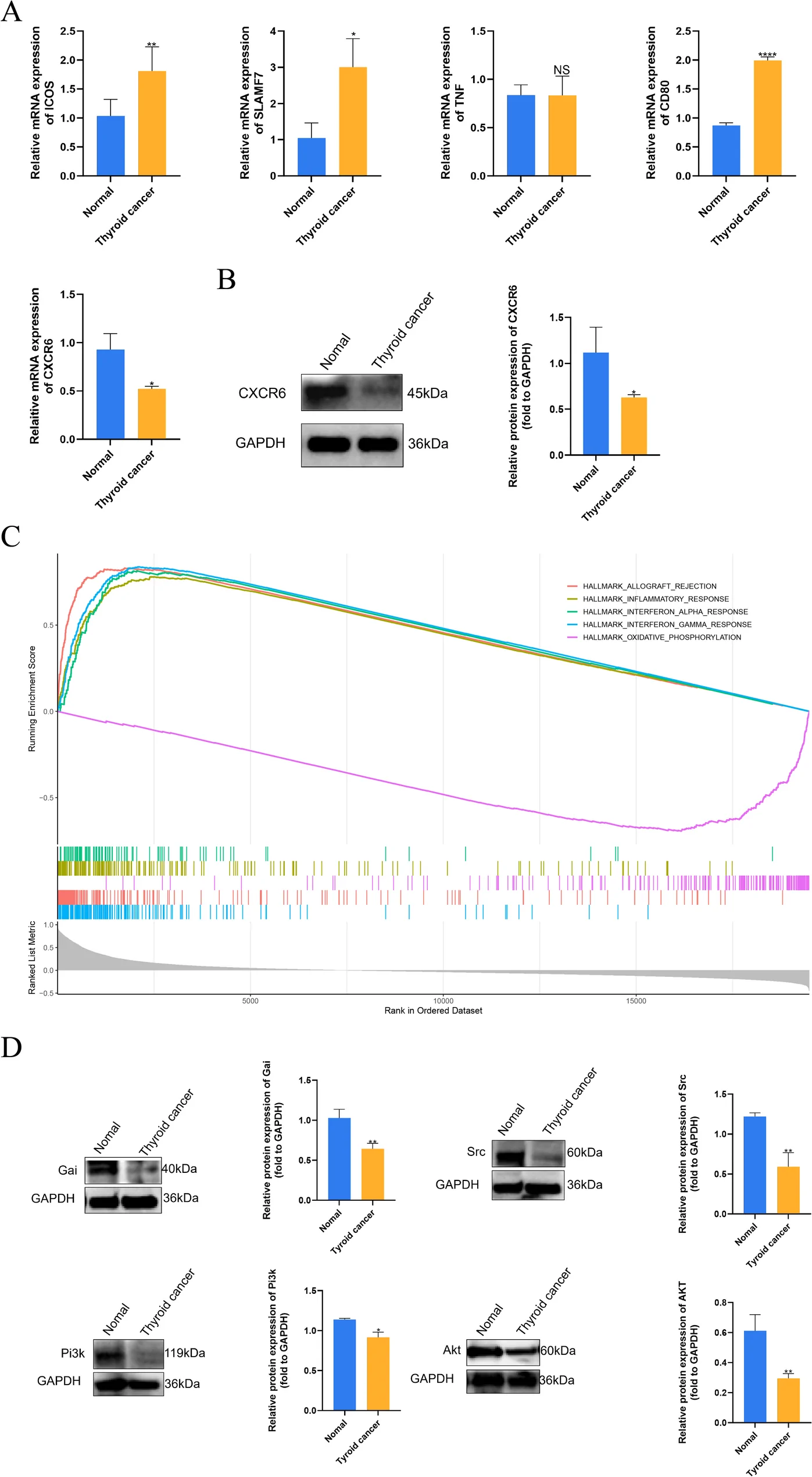
CXCR6 regulated THCA through Gαi, Src, PI3K-AKT pathways. **A **The expression of prognostic genes between control and THCA tissue samples. **B** The expression of CXCR6 between control and THCA samples at protein level. **C **Gene set enrichment analysis of CXCR6. **D** The expression of protein for CXCR6-related pathways. ns, no significance; *P < 0.05, **P < 0.01, ****P < 0.0001

To further investigate expression patterns of prognostic genes, we performed RT-qPCR, which revealed a decrease in CXCR6 levels in THCA samples (Fig. 6A).

Consistent with single-cell transcriptomic results showing dominant CXCR6 expression restricted to T cell populations (Fig. S5), this bulk-level downregulation largely reflects altered CXCR6 abundance within tumor-infiltrating T cells rather than follicular thyroid tumor cells. This finding was consistent with our bioinformatics analysis, prompting us to designate CXCR6 as a key ICD-related gene. Western blot analysis also confirmed that CXCR6 protein levels were significantly reduced in THCA samples (Fig. 6B). Subsequently, we conducted GSEA on CXCR6, identifying 25 enriched pathways (Fig. 6C). Notably, CXCR6 was linked to the inflammatory response, with positive correlations observed. So we also examined key targets of inflammatory response, focusing on G protein-coupled receptors (GPCRs) and their downstream pathways (Src and PI3K-AKT pathways) at protein level. The expression levels of Gαi, Src, PI3K, and AKT were all significantly lower in THCA samples (Fig. 6D), suggesting that the regulation function of CXCR6 was associated with GPCRs and PI3K-AKT pathways to mediate ICD-associated inflammatory response and cytotoxic T lymphocyte recruitment. Notably, only total PI3K and total AKT protein were detected in our Western blot assays, without measurement of phosphorylated active variants. Pathway oncogenic activity relies on phosphorylation status rather than total protein quantity. The observed reduction in total Gαi, PI3K and AKT mainly stems from significantly decreased CXCR6-expressing T cell infiltration in THCA lesions, which weakens upstream GPCR inflammatory signaling. This tissue-level reduction of total pathway proteins derived from immune compartments does not conflict with the well-documented hyperphosphorylation of PI3K-AKT within thyroid tumor epithelial cells reported in previous literature [33, 34]. In addition, CXCR6 expression level alone cannot fully mirror the effector activity of Cytotoxic T Lymphocytes (CTLs); complementary T cell functional markers will be examined via multiplex Immunohistochemistry (IHC) in follow-up work to quantify ICD activity directly.

## Discussion

THCA is a common endocrine tumor with a rising incidence, though its molecular regulatory mechanisms are not yet fully understood. ICD, an immune-related process, has been implicated in THCA progression. Through integrated analysis of single-cell and bulk transcriptomic data, we identified T cells as the key cell type associated with ICD in THCA. We successfully screened five critical prognostic genes, including CXCR6, and constructed a prognostic model with strong predictive performance.

Further mechanistic investigations revealed that CXCR6 may influence THCA progression by regulating Gαi/Src and PI3K-AKT signaling pathways. These findings provide new evidence for understanding the role of ICD in THCA at single-cell resolution and offer potential biomarkers and therapeutic targets for prognosis assessment and targeted treatment of this disease.

Single-cell analysis identified T cells as the key cell type with the highest ICDRG scores in THCA. As central players in anti-tumor immunity, T cell dysfunction or exhaustion is closely related to tumor immune escape [35]. Previous studies have shown that T cell infiltration in the THCA microenvironment is associated with better prognosis, and enhancing T cell-mediated anti-tumor immunity is a promising therapeutic strategy [36, 37]. In this study, pseudotime analysis showed that T cells in THCA samples had more differentiation states, and cell communication analysis indicated enhanced interactions between T cells and other cell types such as follicular cells and fibroblasts in THCA. These results suggest that T cells may regulate the tumor immune microenvironment through ICD-related pathways, thereby participating in the regulation of THCA progression. The enrichment of ICDRGs in T cells further indicates that T cells may be the main effector cells mediating ICD in THCA, which is consistent with the role of T cells in anti-tumor immunity reported in previous studies.

Based on the pivotal role of T cells in the ICD-associated microenvironment of THCA, we further identified five ICD-associated genes at the molecular level that are closely associated with patient prognosis. Among them, CXCR6 was identified as a key gene due to its consistent downregulation in both bioinformatics analysis and clinical samples. CXCR6 is a G protein-coupled receptor (GPCR) redominantly expressed on T cell clusters as validated by our single-cell data, which plays an important role in immune cell recruitment and activation and serves as a biomarker linked to ICD signature. Previous studies have shown that CXCR6 is downregulated in various cancers and is associated with poor prognosis [38]. In this study, RT-qPCR and Western blot confirmed that the bulk-level CXCR6 reduction observed in THCA primarily reflects impaired T cell infiltration rather than intrinsic tumor cell phenotypes. GSEA analysis showed that CXCR6 was positively correlated with the inflammatory response core to ICD, and Western blot results indicated that the expression levels of Gαi (a key target of GPCRs), Src, and PI3K-AKT pathway-related proteins were significantly lower in THCA samples. The PI3K-AKT pathway is a classic signaling pathway regulating cell proliferation, survival, and migration, and its abnormal activation is common in THCA. Combined with the key role of T cells identified in this study, we speculate that CXCR6 may regulate ICD-associated inflammatory response and the activation of the PI3K-AKT pathway through Gαi/Src signaling to facilitate CTL recruitment, thereby affecting the anti-tumor function of T cells and participating in the progression of THCA. This mechanism is supported by previous studies showing that CXCR6 can regulate the migration and activation of T cells by binding to its ligand CXCL16, thereby affecting the tumor immune microenvironment [38]. In addition, other prognostic genes also play important roles in THCA: ICOS and CD80 are important co-stimulatory molecules involved in T cell activation [39, 40]; SLAMF7 is involved in the activation and proliferation of immune cells [41]; TNF is a key pro-inflammatory cytokine regulating immune responses [42, 43]. The abnormal expression of these genes may synergistically regulate the ICD process and anti-tumor immune response in THCA, which is consistent with the enrichment results of T cell activation and cytokine-cytokine receptor interaction pathways in the enrichment analysis of DE-ICDRGs. Beyond modulating immune inflammatory signaling, CXCR6 participates in tumor metastatic progression across multiple malignancies.

Mechanistically, CXCR6 depletion may facilitate THCA invasive and metastatic phenotypes by regulating core metastatic mediators including MMP1, MMP2 and TGF-β, which remodel extracellular matrix and establish a pro-metastatic microenvironment [44–48]. Further in vitro and in vivo functional assays will verify the direct regulatory association between CXCR6 and these metastasis-related molecules in THCA.

Immune infiltration analysis showed significant differences in 8 immune cell types (including naive B cells, plasma cells, CD8 + T cells, and monocytes) between the high-risk and low-risk groups. Among them, SLAMF7 had the highest positive correlation with plasma cells and the highest negative correlation with monocytes. Plasma cells secrete antibodies and participate in humoral immunity, and their infiltration in THCA has been confirmed to be associated with better prognosis [49]. In contrast, monocytes can differentiate into tumor-associated macrophages (TAMs) that promote tumor progression [50, 51]. The positive correlation between SLAMF7 and plasma cells suggests that SLAMF7 may enhance humoral immunity by promoting plasma cell function, thereby inhibiting THCA progression; the negative correlation with monocytes may indicate that SLAMF7 can inhibit the recruitment of monocytes or their differentiation into TAMs, further regulating the tumor immune microenvironment. Immunotherapeutic efficacy is largely governed by comprehensive TME components including infiltrated immune subsets, secreted cytokines and tumor-derived suppressive mediators [52–54]. Our CXCR6-based ICD signature reflects functional status of anti-tumor T cells within THCA TME; downregulated CXCR6 impairs inflammatory cytokine signaling and cytotoxic T lymphocyte recruitment, which may further reduce clinical response to immune checkpoint blockade in THCA patients. These results highlight the important role of prognostic genes in regulating the immune microenvironment of THCA, which is consistent with the core role of ICD in linking cell death and immune response.

Complementary Scissor single-cell dissection resolved intratumoral heterogeneous gene expression linked to patient survival and supplied candidate genes for our ICD signature screening; drug sensitivity and BRAF mutation analyses further extended the translational value of the risk model by identifying risk-stratified differential responses to BRAF-targeted therapy, which jointly reinforce the clinical utility of our ICD-related prognostic signature beyond pure prognostic prediction.

The innovation of this study lies in the systematic integration of scRNA-seq and bulk RNA sequencing data to screen ICD-related prognostic genes in THCA, and the construction of a reliable prognostic model using multiple machine learning algorithms. We identified CXCR6 as a key gene and clarified its role in regulating THCA progression through the Gαi, Src, and PI3K-AKT pathways, filling the research gap regarding the role of CXCR6 in ICD-related THCA progression.

However, this study also has several limitations that need to be acknowledged. First, the normal thyroid controls from the TCGA cohort were only labeled as non-tumor per routine histology, without dedicated staining to exclude subclinical thyroid inflammation such as occult hypothyroidism. Elevated mast cell infiltration detected in these public normal samples may introduce baseline inflammatory bias in immune cell and ICD gene comparisons. Meanwhile, the scRNA-seq dataset GSE191288 only contains a single normal control sample, lacking sufficient replicates for robust analysis. To partially offset this drawback, we included 6 pairs of self-collected clinical THCA and matched adjacent normal tissues, all pathologically confirmed free of inflammatory infiltration; consistent CXCR6 expression trends in these independent samples validated our core bioinformatics findings. In addition, bulk RNA and protein detection in our clinical tissue samples cannot distinguish CXCR6 expression across immune and tumor cell compartments, and multiplex IHC staining for cell-type localization and T cell functional markers was not implemented in the current manuscript due to limited sample resources and experimental schedule.

Second, we only validated gene and pathway expression at the tissue level, and the precise molecular mechanism whereby CXCR6 modulates THCA progression via Gαi/Src/PI3K-AKT signaling requires further in vitro and in vivo functional verification. The current Western blot experiments only quantitatively determined the total levels of PI3K and AKT by using batch-organized lysis buffers. Lower total protein stems from impaired T cell infiltration, while PI3K-AKT activation in thyrocytes is driven by phosphorylation independent of total protein levels. Follow-up cellular and animal experiments will include phosphorylated PI3K/AKT detection to separately evaluate pathway activity in tumor cells and immune subsets. Notably, metabolic remodeling in thyroid tumor cells may disturb endogenous GAPDH expression and introduce minor quantification bias for Western blot analysis. However, multiple previous studies have verified GAPDH as a stable internal reference for protein quantification in thyroid tumor and matched normal tissues, and this normalization strategy has been widely adopted in thyroid cancer research [55–57].

Unified experimental operations and consistent target protein expression trends across paired clinical samples ensured the credibility of our core comparative results; total protein staining or other stable housekeeping proteins will be used for loading correction in our follow-up cellular and animal functional assays to improve the robustness of protein quantification results. Third, our in-house clinical validation cohort has a relatively small sample size, and the prognostic performance of CXCR6 and other ICD-related genes needs validation in larger independent patient cohorts. In future work, we will collect additional pathologically confirmed inflammation-free normal thyroid tissues for single-cell sequencing to eliminate inflammatory background interference and conduct functional experiments to dissect the downstream regulatory network of CXCR6 in THCA. Fourthly, partial TCGA-THCA samples lacked complete records of histological subtype and postoperative treatment regimens, which limited our ability to adjust these parameters in multivariate Cox regression analyses when evaluating the independent prognostic value of our risk signature. We recognize the incomplete clinical covariate information of the public TCGA cohort as an inherent limitation of the present study. Another limitation is that we did not conduct secondary subclustering of the T cell population to distinguish CD8^+^ cytotoxic T cells, CD4^+^ helper T cells, Tregs and exhausted T cell subsets. The core purpose of this work was to identify ICD-associated prognostic genes with clinical predictive value for THCA patients, so we focused on global ICDRG score comparisons across major cell lineages rather than resolving T cell intrasubset heterogeneity. Further single-cell subclustering analysis of T cell subtypes paired with subtype-specific ICD signature quantification will be implemented in our subsequent research to clarify which T cell subpopulation dominates ICD regulation in the THCA tumor microenvironment.

## Conclusion

Through multi-omics analysis, this study identified T cells as the key cell type mediating ICD in THCA and screened five ICD-related prognostic genes (CXCR6, ICOS, SLAMF7, CD80, and TNF). Among them, T cell-enriched CXCR6 was confirmed as a key ICD-related gene significantly downregulated in THCA, and its regulatory effect on THCA progression is associated with the Gαi, Src, and PI3K-AKT pathways governing immune recruitment and inflammatory response. In addition, the prognostic model constructed based on these genes has good predictive ability for the survival of THCA patients, and these prognostic genes are closely related to the regulation of the tumor immune microenvironment. These findings not only clarify the role and molecular mechanism of ICD-related genes in THCA progression but also provide new potential biomarkers and therapeutic targets for the prognostic assessment and treatment of THCA.

## Supplementary Information

Supplementary Material 1: Fig. S1 Single-cell analysis. Fig. S2 Kaplan-Meier (KM) survival analysis and pan-cancer expression of prognostic genes. Fig. S3 KM survival analysis for DFI, DSS, OS, and PFI of pan-cancer in different prognostic gene high and low expression groups. Fig. S4 Cox regression analysis. Fig. S5 Expression map of CXCR6 in single-cell analysis. Table S1 The list of immunogenic cell death-related genes (ICDRGs). Table S2 Summary table of the classic marker genes for each annotated cell subtype. Table S3 The detailed information of antibodies used in Western blot.

## Data Availability

The bulk RNA-sequencing data analyzed in this study were obtained from the TCGA database (https://portal.gdc.cancer.gov/, project ID: TCGA-THCA). The single-cell RNA-sequencing data were acquired from the Gene Expression Omnibus (GEO) database under accession number GSE191288. The 32 immunogenic cell death-related genes were derived from a previously published study. All data used in this study are publicly available and can be accessed from the corresponding databases.

## Abbreviations

AUC: Area under the curve
DAMPs: Damage-associated molecular patterns
DEGs: Differentially expressed genes
GEO: Gene expression omnibus
GO: Gene ontology
GPCR: G protein-coupled receptor
GSEA: Gene set enrichment analysis
ICD: Immunogenic cell death
ICDRGs: ICD-related genes
KEGG: Kyoto encyclopedia of genes and genomes
PTC: Thyroid papillary carcinoma
RT-qPCR: Real-time Quantitative Polymerase Chain Reaction
TCGA-THCA: THCA-related bulk RNA
THCA: Thyroid cancer
