# Supplementary material for: CXCR6 and its correlated G protein-coupled receptors and PI3K-AKT pathways were associated with the progression of thyroid cancer
Source: BMC Cancer. 2026 Jul 10;26:1110. doi: 10.1186/s12885-026-16513-1 (PMC13584473; doi:10.1186/s12885-026-16513-1)

Figure 6B CXCR6

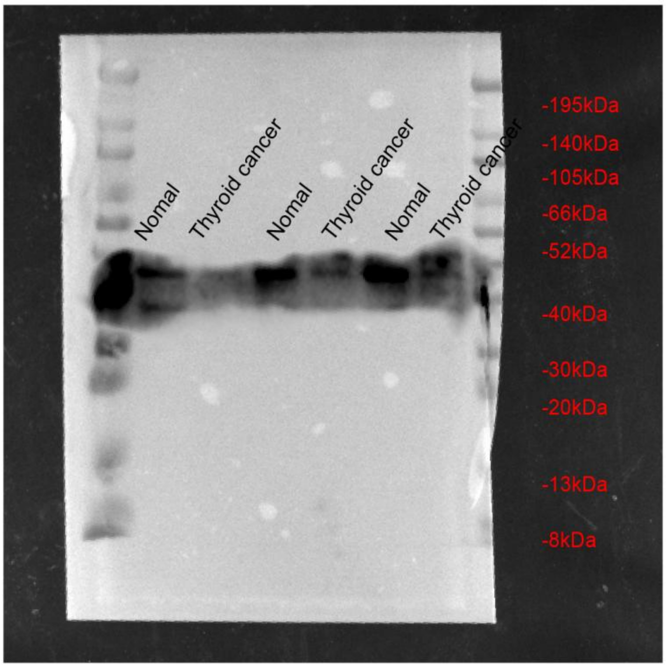

Figure 6B CXCR6-GAPDH

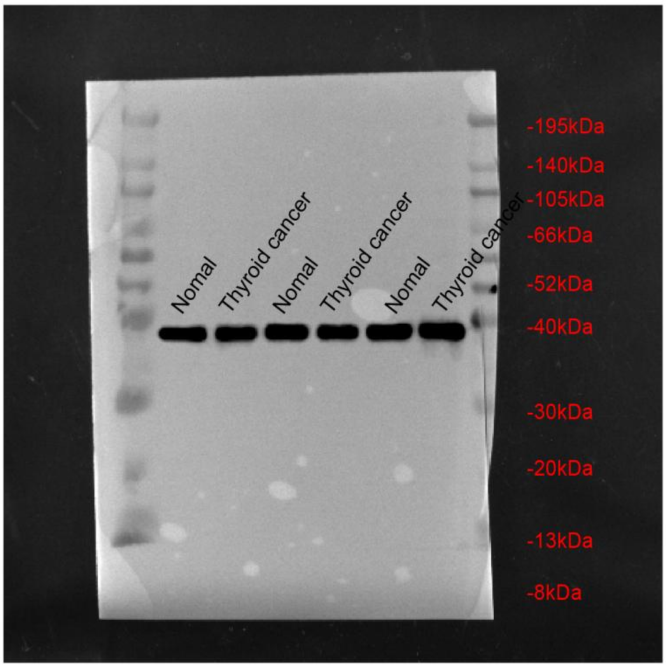

Figure 6D Gai

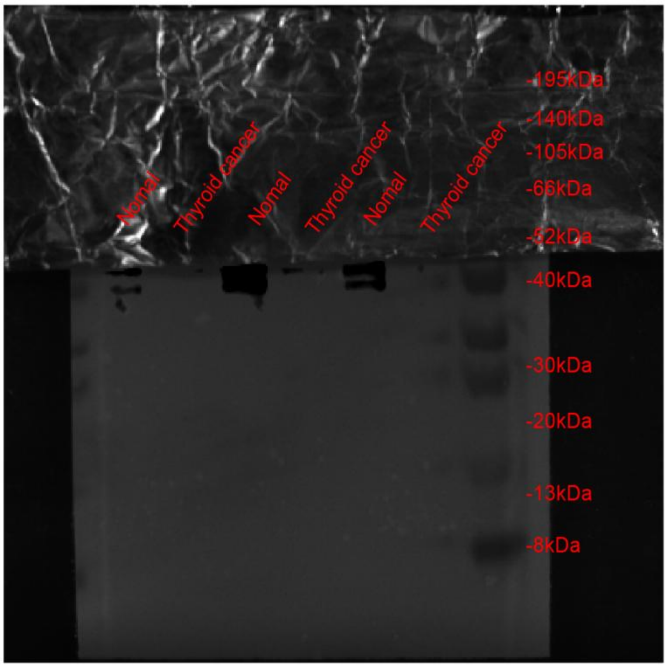

Figure 6D Gai-GAPDH

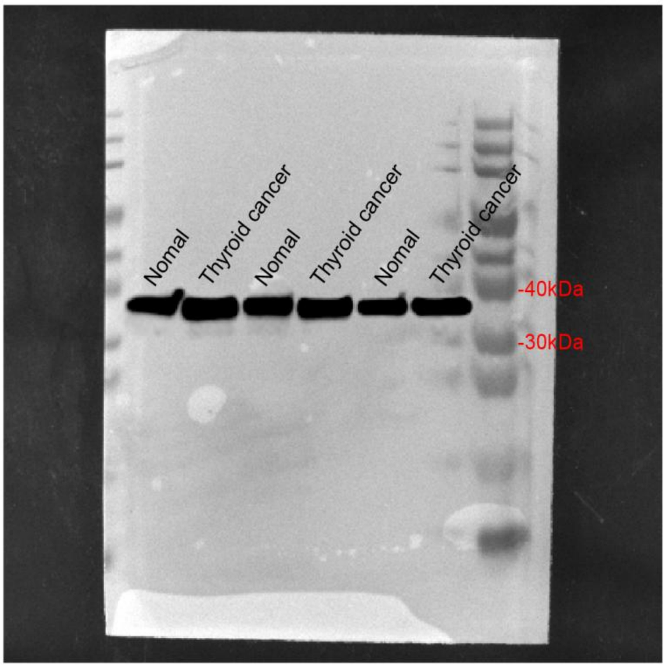

Figure 6D Src

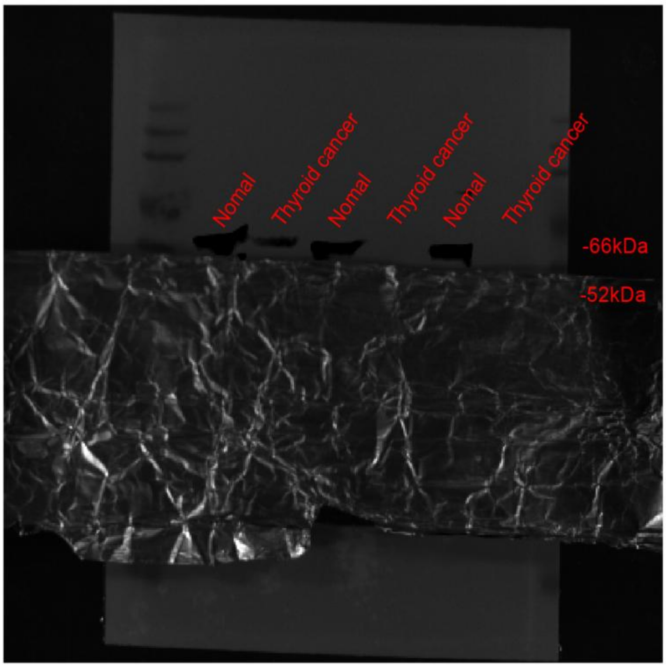

Figure 6D Src-GAPDH

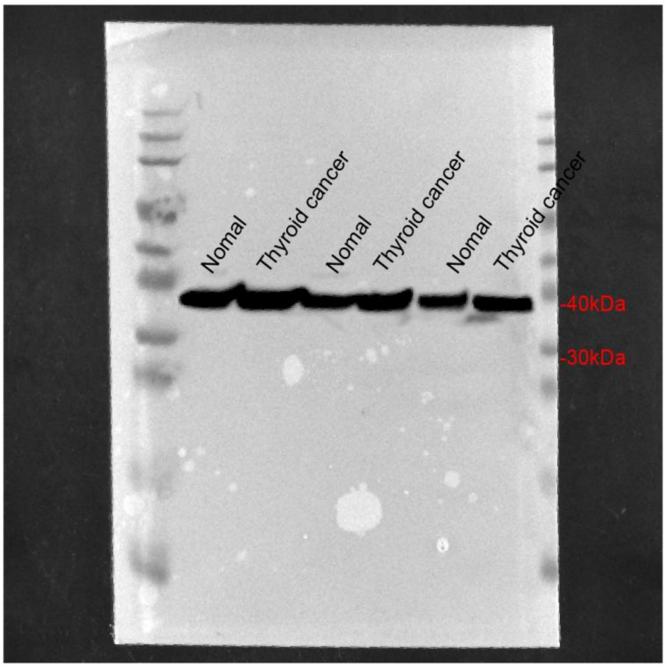

Figure 6D Pi3k

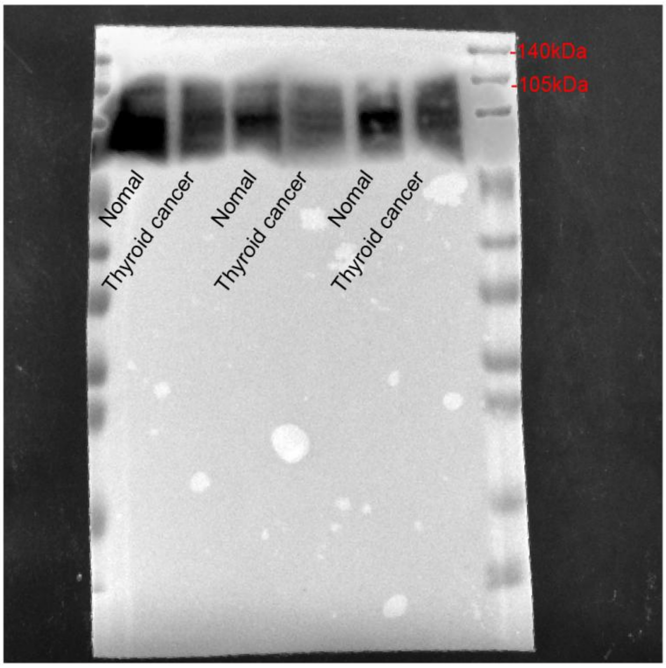

Figure 6D Pi3k-GAPDH

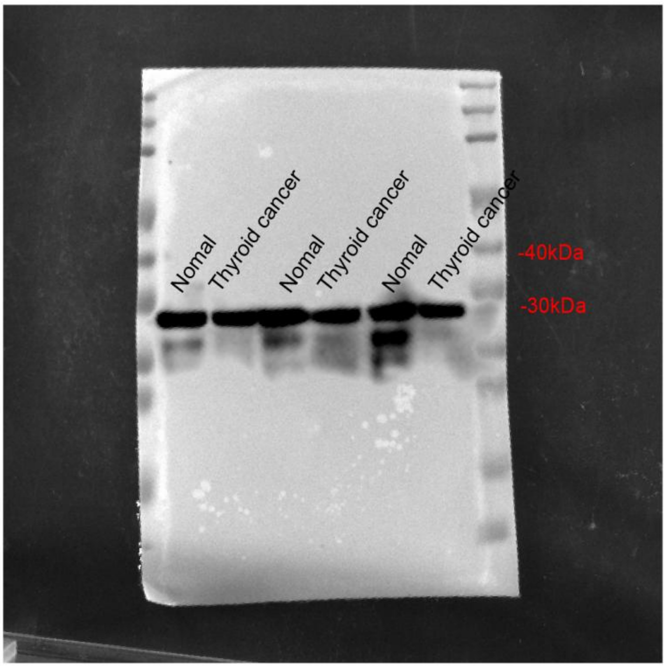

Figure 6D Akt

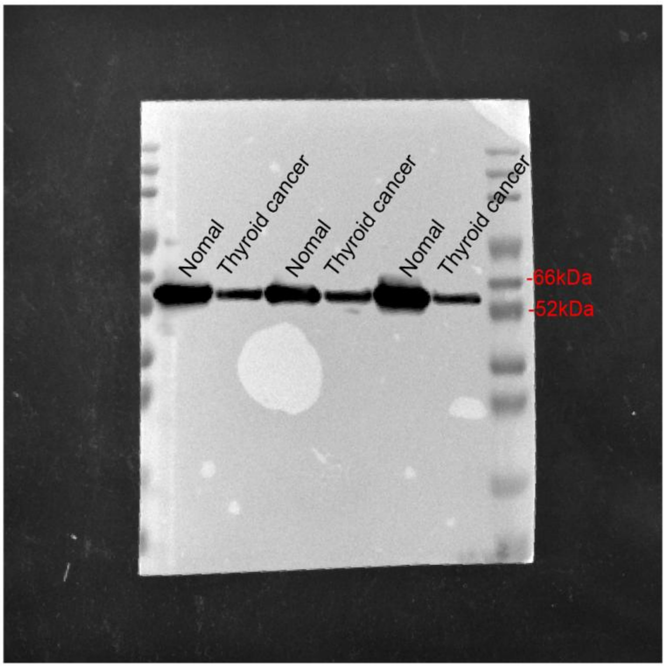

Figure 6D Akt-GAPDH

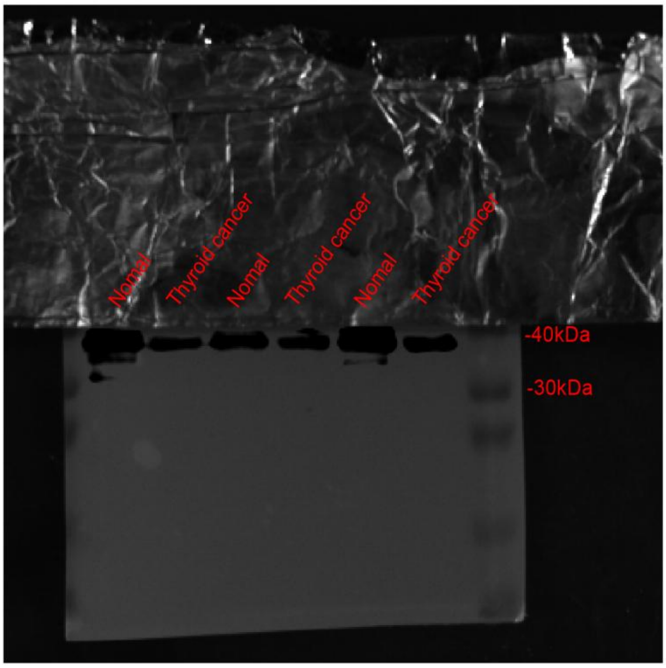

Supplement: Supplementary file 1 [file 12885_2026_16513_MOESM1_ESM.zip › supplementary file.pdf]
