# Supplementary material for: CXCR6 and its correlated G protein-coupled receptors and PI3K-AKT pathways were associated with the progression of thyroid cancer
Source: BMC Cancer. 2026 Jul 10;26:1110. doi: 10.1186/s12885-026-16513-1 (PMC13584473; doi:10.1186/s12885-026-16513-1)
Supplement: Supplementary file 1 [file 12885_2026_16513_MOESM1_ESM.zip › Table S3.docx]

**Table S3** The detailed information of antibodies used in Western blot

| **Antibodies** | **Manufacturer** | **Number** | **Dilution ratio** |
| --- | --- | --- | --- |
| GAPDH Rabbit pAb | Abclonal | AC001 | 1: 10000 |
| CXCR6 Rabbit pAb | Abclonal | CXCR6 Rabbit pAb | 1: 1000 |
| GNAI1 Rabbit pAb（Gai） | ABclonal | A8844 | 1: 1000 |
| Src Rabbit pAb | ABclonal | A0324 | 1: 1000 |
| AKT1 Recombinant Rabbit Monoclonal Antibody (ST05-09) | Invitrogen | MA5-41138 | 1: 5000 |
| PIK3CD Recombinant Rabbit Monoclonal Antibody (JM83-24) | Invitrogen | MA5-32747 | 1: 500 |
| Goat anti-Rabbit IgG (H+L) Secondary Antibody, HRP | Invitrogen | 31460 | 1: 5000 |
