# Supplementary material for: CXCR6 and its correlated G protein-coupled receptors and PI3K-AKT pathways were associated with the progression of thyroid cancer
Source: BMC Cancer. 2026 Jul 10;26:1110. doi: 10.1186/s12885-026-16513-1 (PMC13584473; doi:10.1186/s12885-026-16513-1)
Supplement: Supplementary file 1 [file 12885_2026_16513_MOESM1_ESM.zip › supplementary file.docx]

Figure 6B CXCR6 - GAPHD

Figure 6D Gai- GAPHD

Figure 6D Src- GAPHD

Figure 6D Pi3k- GAPHD

Figure 6D Akt- GAPHD
