# Supplementary material for: CXCR6 and its correlated G protein-coupled receptors and PI3K-AKT pathways were associated with the progression of thyroid cancer
Source: BMC Cancer. 2026 Jul 10;26:1110. doi: 10.1186/s12885-026-16513-1 (PMC13584473; doi:10.1186/s12885-026-16513-1)
Supplement: Supplementary file 1 [file 12885_2026_16513_MOESM1_ESM.zip › Supplementary Figure 1-3.docx]

**Fig. S1** Single-cell analysis. (**A**) Quality control for single cell RNA sequencing (scRNA-seq) dataset. (**B**) The elimination of doublets. (**C**) The expression of immunogenic cell death-related genes (ICDRGs) during key ell type differentiation.

**Fig. S2** Kaplan-Meier (KM) survival analysis and pan-cancer expression of prognostic genes. (**A**) KM survival curves for prognostic gene high and low expression groups. (**B**) The expression of prognostic genes between pan-cancer and control samples. **P* < 0.05, ***P* < 0.01, ****P* < 0.001, *****P* < 0.0001.

**Fig. S3** KM survival analysis for DFI, DSS, OS, and PFI of pan-cancer in different prognostic gene high and low expression groups.
